# Comparisons of efficacy and safety between preserved and preservative-free brimonidine tartrate in glaucoma and ocular hypertension: a parallel-grouped, randomized trial

**DOI:** 10.1038/s41598-023-31726-1

**Published:** 2023-04-07

**Authors:** Ko Eun Kim, Chang Kyu Lee, Jonghoon Shin, Yuri Kim, Seungsoo Rho

**Affiliations:** 1 Department of Ophthalmology, Asan Medical Center, University of Ulsan College of Medicine, Seoul, Republic of Korea; 2 Department of Ophthalmology, Ulsan University Hospital, University of Ulsan College of Medicine, Ulsan, Republic of Korea; 3 Department of Ophthalmology, Pusan National University Yangsan Hospital, Pusan National University School of Medicine, Yangsan, Republic of Korea; 4 Research Institute for Convergence of Biomedical Science and Technology, Pusan National University Yangsan Hospital, Yangsan, Republic of Korea; 5 Department of Ophthalmology, CHA Bundang Medical Center, CHA University, 59 Yatap-ro, Bundang-gu, Seongnam-si, Gyeonggi-do 13496 Republic of Korea

**Keywords:** Glaucoma, Randomized controlled trials

## Abstract

This multicenter (four institutions), randomized, investigator-masked, parallel-group clinical trial evaluated and compared the efficacy and safety of preservative-free and preserved brimonidine tartrate 0.15% in open-angle glaucoma and ocular hypertension. Sixty eyes of 60 patients with intraocular pressure (IOP) ≥ 15 mmHg diagnosed with open-angle glaucoma or ocular hypertension were randomized to preserved (n = 31) and preservative-free (n = 29) brimonidine groups. The enrolled eyes received brimonidine monotherapy three times daily. Main outcome measures were corneal/conjunctival staining score, ocular surface disease index, patient satisfaction score, drug tolerance, and drug adherence rate 12 weeks post first administration. Secondary outcome measurements included visual acuity, IOP, drug tolerance, tear-film break-up time, hemodynamic changes including blood pressure and heart rates, and ocular adverse events. After 12 weeks, both preserved and preservative-free groups showed similar IOP reduction, corneal and conjunctival staining scores, drug tolerance, and adherence rates. The preservative-free group showed significantly better tear-film break-up time and higher patient satisfaction regarding drug use and management. Systolic and diastolic blood pressure reductions during the 12 weeks were significantly lower in the preserved group than in the preservative-free group. Preservative-free brimonidine tartrate showed comparable efficacy and safety, better corneal tear film stability, and patient satisfaction than preserved brimonidine.

## Introduction

Brimonidine tartrate is a selective *a*2-adrenergic agonist used to lower intraocular pressure (IOP) for the treatment of glaucoma and ocular hypertension^1,2^. It reduces aqueous humor production by causing vasoconstriction in the anterior segment and continues to lower IOP by increasing uveoscleral outflow^3,4^. Brimonidine tartrate has long been used and is commonly considered a first-line IOP lowering eyedrop for glaucomatous eyes. A comparative study demonstrated that it was the most effective neuroprotective drug^5^, although with risks of systemic complications such as blood pressure reduction and dry mouth^6^.

Preservative-free IOP-lowering medication packaged in single unit-dose pipettes with fewer additives has been gradually substituted for a large portion of preserved medications. Preservative-free medication is easy to use, allows for easy counting of doses, and, most importantly, is less toxic to the cornea, resulting in reduced irritation and dry eye^7,8^. Currently, Alphagan P^®^ (Allergan Inc., Irvine, CA, USA), the commonly available preserved type of brimonidine tartrate, contains the preservative Purite^®^, a stabilized oxychloro complex oxidizing microbial cellular components, which is expected to have no significant effect on human ocular tissue, as it breaks down upon contact with the air during instillation into natural tear components such as sodium and chloride ions, oxygen, and water^9^. While laboratory data have demonstrated the reduced corneal toxicity of Purite compared to other preservatives^10,11^, evidence is scarce regarding these effects in real clinical settings. Recently, a preservative-free brimonidine has been developed to reduce preservative-induced complications and increase patient adherence. Given the long-term use of glaucoma medication, it is important to understand how the constituents of each formulation may affect the ocular surface, symptoms, systemic parameters, and overall patient satisfaction and compliance.

The main purpose of this study was to prospectively compare the efficacy and safety of preservative-free brimonidine tartrate 0.15% to preserved brimonidine tartrate 0.15% with regard to corneal surface assessment, IOP reduction, safety, and adherence rates in patients with open-angle glaucoma or ocular hypertension. Moreover, to evaluate the potential benefits of preservative-free brimonidine, ocular and systemic parameters, and questionnaire-based patient satisfaction and drug tolerance scores were analyzed.

## Methods

### Study design

The present multicenter, randomized, open-label, investigator-masked, parallel-group clinical trial investigated the efficacy and safety of preservative-free and preserved brimonidine in patients with open-angle glaucoma or ocular hypertension. The involved institutions were CHA Bundang Medical Center, Pusan National University Yangsan Hospital, Ulsan University Hospital, and Nowon Eulji Medical Center. This study was approved by the institutional review boards of CHA Bundang Medical Center, Pusan National University Yangsan Hospital, Ulsan University Hospital, and Nowon Eulji Medical Center and adhered to the tenets of the Declaration of Helsinki. This study conformed to CONSORT 2010 guidelines (www.consort-state.org) and was registered at https://www.clinicaltrials.gov on 23/11/20200 (NCT04647461).

The participants were enrolled in four institutions from June 2019 to January 2021. The participants were fully informed and voluntarily provided written consent before screening and were randomized into two groups after providing agreement; namely, the unit-dose preservative-free brimonidine (Bridin-T^®^; Hanlim Pharm., Seoul, South Korea) and multi-dose preserved brimonidine (Alphagan P^®^) groups. We performed a centralized and automated allocation in a blind manner using an interactive web-based randomization system (IWRS, TnW software Ltd., Seoul, South Korea) running 24 h during the whole study period. All the patient information and variables were uploaded to a web-based electronic case report form (ver 1.0, http://www.ecrf.kr, TnW software Ltd., Seoul, South Korea). The same external package was used for each group’s investigation product for masking, as the investigators were blinded throughout the study period. The patients were instructed to instill either preservative-free or preserved brimonidine three times daily from day 0 and to visit the clinic at 4 and 12 weeks. At both 4-and 12-week visits, the patients underwent follow-up measurements at 10 AM ± 1 h and instilled the eyedrops thereafter.

### Patients

All patients underwent best-corrected visual acuity (BCVA) and IOP measurement by Goldmann applanation tonometry, as well as central corneal thickness (CCT) measurement. They also underwent fundus photography, red-free photography, spectral-domain optical coherence tomography, and visual field examination using a Humphrey Field Analyzer (Carl Zeiss Meditec, Dublin, CA, USA). The glaucomatous changes were determined as typical optic disc/retinal nerve fiber layer changes corresponding to reproducible glaucomatous visual field defects by glaucoma specialists in each institution.

The inclusion criteria were patients aged ≥ 19 years with open-angle glaucoma or ocular hypertension with an untreated IOP ≥ 15 mmHg and < 40 mmHg as measured by Goldmann applanation tonometry by a masked, assigned examiner at the screening visit after a proper washout period. All patients receiving IOP-lowering treatment underwent a 4-week washout period except for those using cholinergic eye drops and carbonic anhydrase inhibitors, in which the washout period was 5 days. The open angles were determined by gonioscopy. If both eyes were eligible, the one with higher IOP was enrolled; however, if both IOPs were the same, the right eye was enrolled.

The exclusion criteria were BCVA less than 20/80, extreme CCT measurement (e.g., outside the range of 470–591 µm), history of angle closure or primary angle closure, prior glaucoma surgery, prior or currently active inflammatory disease, prior ocular trauma, history of intraocular surgery other than simple cataract surgery, retinal laser treatment, any non-glaucomatous optic neuropathy, any macular or retinal disease affecting the disc/retinal nerve fiber layer and the visual field, systemic disorders, and pregnancy. Patients with prior lacrimal punctal occlusion procedures in the past months or in requiring topical treatment (e.g., hyaluronic acid, cyclosporine, diquafosol, autologous serum) for severe dry eye disease were excluded. Patients unable to make voluntary decisions were also excluded from enrollment.

### Study methods

The conjunctival staining scores were evaluated according to the National Eye Institute scale (0–3) with fluorescein staining after dividing the conjunctiva into six areas. The corneal staining scores were determined according to the Oxford grading system (0–5)^12,13^. The tear-film break-up time (TBUT) was measured twice, with the average values used for analysis. The ocular surface disease index (OSDI^©^) score, a valid and reliable instrument for measuring dry eye disease (normal, mild to moderate, and severe) and the effect on vision-related function^14^, was assessed, in which higher scores represented greater disability and discomfort. The bulbar and limbal hyperemia scores were evaluated using the Efron grading scale (0–4)^15^. Adherence rates (0–100%) were assessed at 4 and 12 weeks using a self-report sheet. The drug tolerance data were acquired using a questionnaire sheet to evaluate the frequency and severity of the symptoms associated with using eye drops, including stinging/burning, sticky sensation, itching, blurring, sandiness/grittiness, dryness, light sensitivity, and pain/soreness. The level of each symptom was graded as 0 (none) to 3 (severe, immensely interfering with the subject's daily life), and the duration of each symptom as 0 (prompt: < 5 min) or 1 (continuous: ≥ 5 min). Systolic BP (SBP), diastolic BP (DBP), and heart rate (HR) were measured with the patients in the sitting position at baseline and at the 4-, and 12- week visits. At the 4- and 12-week visits, the IOP, SBP, DBP, and HR were measured at 10 AM ± 1 h. The IOPs were measured twice, with the average value used for analysis.

### Outcome measures

The primary endpoints were corneal and conjunctival staining score, OSDI score, drug tolerance, and adherence rates at 12-week visits. The secondary efficacy endpoints—corneal and conjunctival staining score, OSDI score at 4-week visits and IOP, TBUT, and bulbar/limbal hyperemia score at the 4- and 12–week visits were analyzed and compared between the groups. For safety assessment, BCVA, SBP, DBP, HR, and physical examination at 4 and 12 weeks and adverse events during the whole study period were analyzed.

### Statistical analysis

This study aimed to evaluate the superiority of preservative-free brimonidine over preserved one in terms of ocular surface conditions. Although there was no equally designed study similar to ours, superiority was concluded if the difference in the hyperemia score was 0.87 or more according to previous studies that evaluated the difference in eye redness in subjects using preserved and preservative-free eyedrops^8,16^. Given that a standard deviation of 1.0 for the hyperemia score was proposed assuming a dropout rate of 30%, a total of 60 patients (30 in each group) should be enrolled to provide 90% power for the superiority calculation. Baseline characteristics were compared between the preserved-free and preserved groups by student’s *t*- or Wilcoxon rank-sum tests for continuous variables and chi-square and Fisher’s exact tests for categorical variables. Intragroup comparisons of serial measurements compared to baseline data were performed using paired *t*- or Wilcoxon signed-rank tests. Intergroup comparisons of continuous outcome measurements at baseline, 4-, and 12-week visits were performed using analysis of covariance after adjusting baseline values and covariates, if needed. All statistical analyses were performed using PASW Statistics for Windows, version 18.0 (SPSS Inc., Chicago, IL, USA). Statistical significance was set at *P* < 0.05. Data are presented as means ± standard deviation, unless otherwise indicated.

## Results

**Figure 1:**
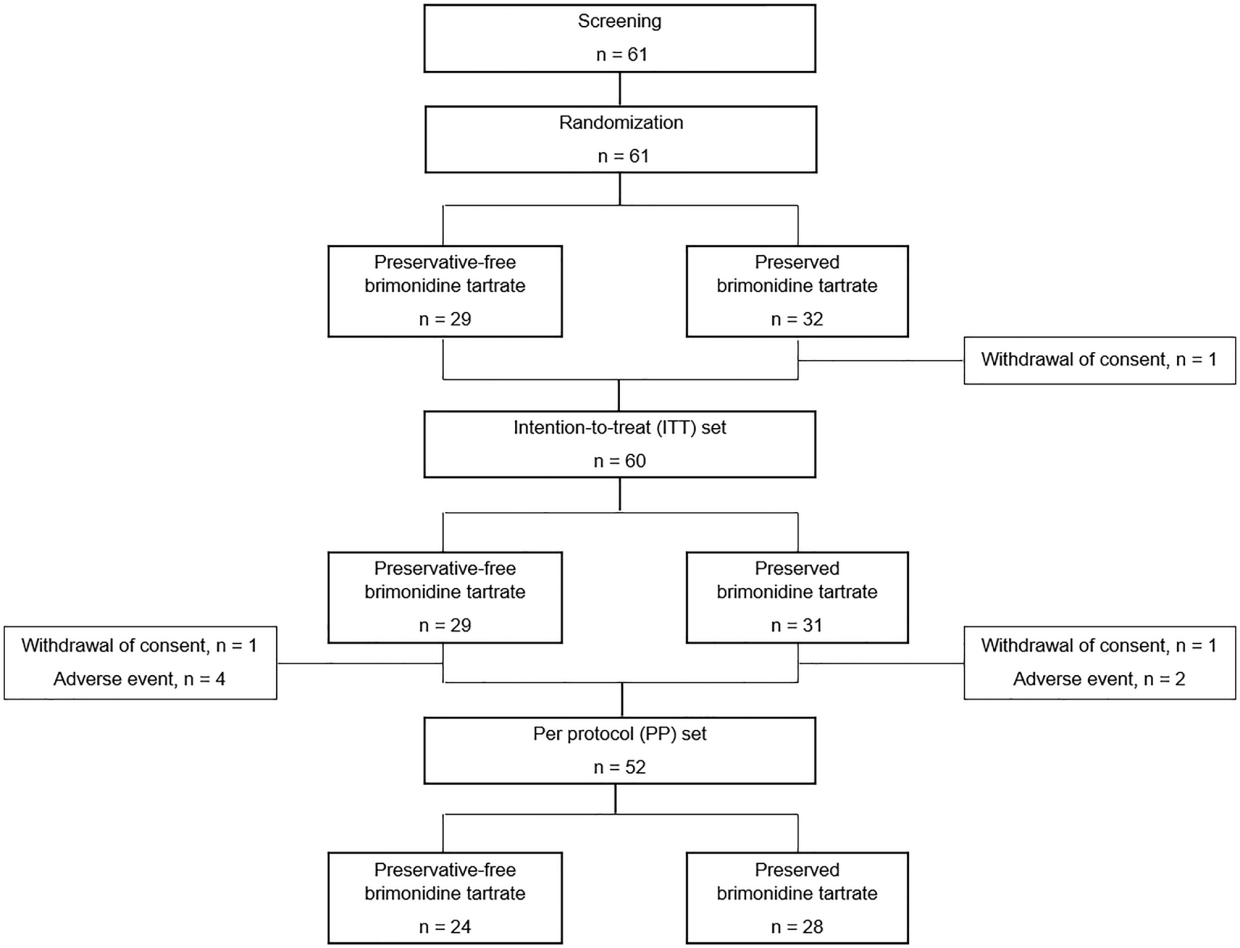
Flow chart of subject enrollment.

Overall, 61 patients were enrolled and randomized into each group (29 preservative-free and 32 preserved; Fig. 1). Before using the eyedrop, one patient (withdrawal of consent) was excluded from the preserved group and consequently, 60 patents comprised the intention-to-treat (ITT) set. Five patients (one withdrawal of consent, four adverse events) were excluded from the preservative-free group and three from the preserved group (one withdrawal of consent, two adverse events). Consequently, 24 and 28 patients were included in the per-protocol (PP) set of preservative-free and preserved groups, respectively. The preservative-free group showed significantly lower mean age, baseline CCT, and SBP compared to the preserved group. The other variables did not differ significantly between the two groups (Table 1).

**Table 1:** Comparison of demographics and baseline characteristics between included patients.

	Preserved (n = 31)	Preservative-free (n = 29)	*P*
Age, years	60.9 ± 11.0	56.7 ± 9.0	**0.037**
Male, n (%)	16 (51.6)	8 (27.6)	0.058
Right eye, n (%)	18 (58.1)	11 (37.9)	0.12
Diagnosis, n (%)			
Glaucoma	28 (90.3)	28 (96.6)	0.654
Ocular hypertension	3 (9.7)	1 (3.4)	
Hypertension, n (%)	15 (51.7)	15 (48.3)	0.796
Height, cm	162.51 ± 9.44	159.88 ± 8.14	0.25
Weight, kg	66.01 ± 12.05	64.04 ± 15.21	0.24
SBP, mmHg	136.80 ± 13.27	126.03 ± 15.79	**0.003**
DBP, mmHg	79.07 ± 11.16	73.90 ± 12.35	0.097
HR, beat per minute	73.40 ± 7.68	78.07 ± 13.22	0.22
BCVA	0.86 ± 0.19	0.93 ± 0.12	0.1498
Intraocular pressure, mmHg	17.90 ± 3.36	17.52 ± 2.60	0.95
Central corneal thickness, µm	551.03 ± 24.59	531.41 ± 23.53	**0.002**
Bulbar hyperemia	1.03 ± 1.02	1.17 ± 0.93	0.44
Limbal hyperemia	0.71 ± 0.90	0.72 ± 0.80	0.76
Corneal staining score	0.71 ± 0.86	0.76 ± 0.79	0.70
Conjunctival staining score	1.28 ± 1.00	1.43 ± 1.07	0.55
Tear film break up time	6.47 ± 2.26	6.46 ± 2.25	0.91
OSDI score	7.84 ± 7.08	8.31 ± 6.70	0.62

*SBP* systolic blood pressure, *DBP* diastolic blood pressure, *HR* heart rate, *BCVA* best corrected visual acuity, *OSDI* ocular surface disease index.

Significant values with *P* < 0.05 are indicated in bold.

### Primary outcomes

**Figure 2:**
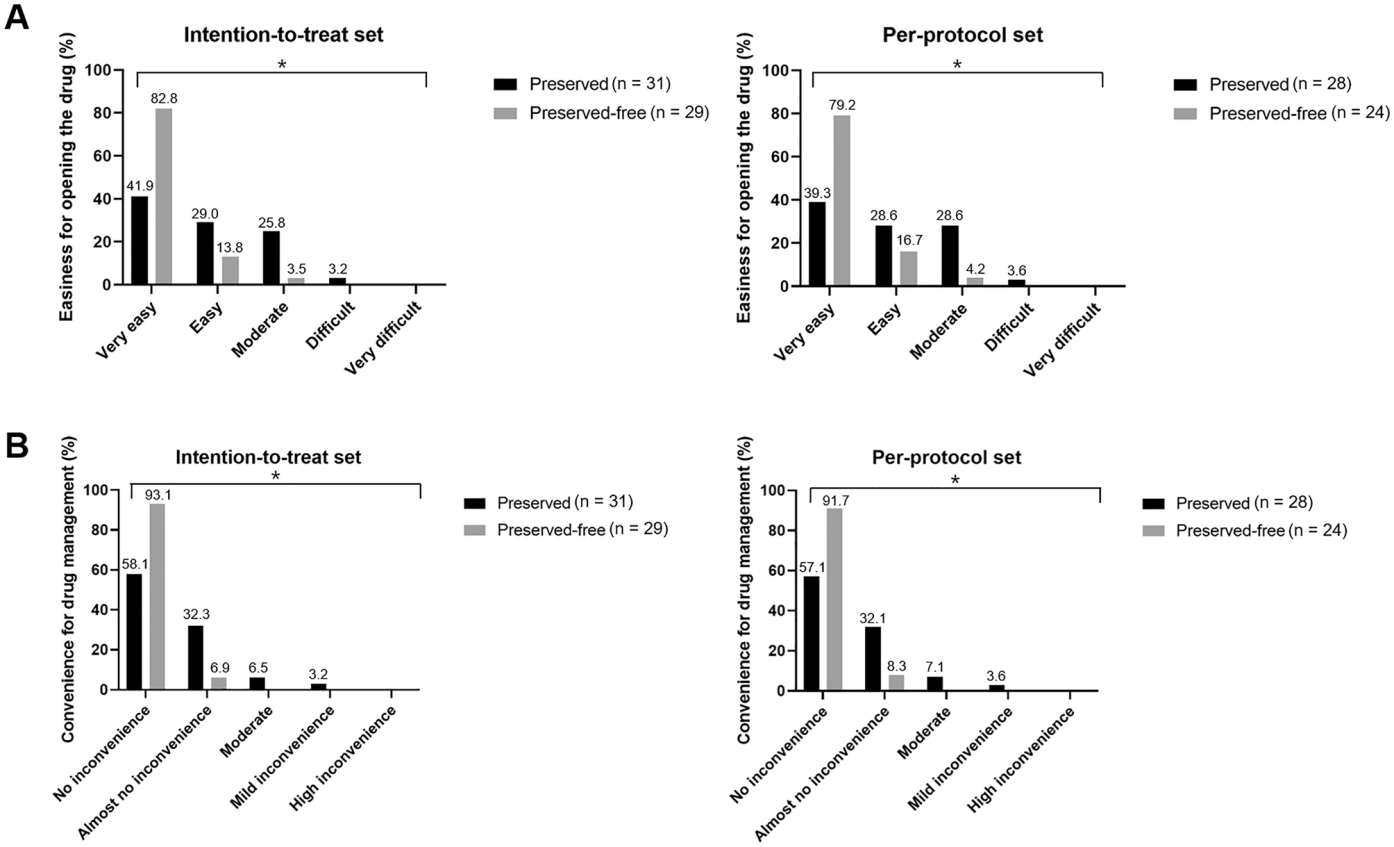
Proportions of patients surveyed regarding (**A**) the ease of opening the drug container and (**B**) the convenience of drug management. In both intention-to-treat (ITT) and per-protocol (PP) sets, significantly higher proportions of patients in the preserved-free group responded that it was very easy to open the drug container compared to preserved group (*P* < 0.001 for the ITT group, *P* = 0.002 for the PP group). In addition, a significantly higher proportion of patients in the preservative-free group reported that the drug container type caused no inconvenience for drug management (*P* = 0.002 for the ITT group and *P* = 0.005 for the PP group).

For analysis of the primary efficacy endpoint, the conjunctival and corneal staining scores, OSDI scores, patient satisfaction, drug tolerance, and adherence rates at 12-week visits were compared between the two groups (Table 2). The corneal/conjunctival staining and OSDI scores did not differ significantly between the preservative-free and preserved groups. The medical adherence rates were also similar between the groups. However, regarding drug satisfaction, higher proportions of patients in the preservative-free group reported that the unit-dose container was easy to open (Fig. 2A) and convenient for drug management (Fig. 2B) compared to the preserved group.

**Table 2:** Primary outcome measurements at 12-week visit.

Variables	Intention-to-treat set			Per-protocol set		
	Preserved (n = 31)	Preservative-free (n = 29)	*P*	Preserved (n = 28)	Preservative-free (n = 24)	*P*
Corneal staining score	0.61 ± 0.67	0.48 ± 0.63	0.43^a^	0.61 ± 0.63	0.54 ± 0.66	0.38^a^
Conjunctival staining score	1.32 ± 0.94	1.30 ± 1.09	0.39^a^	1.25 ± 0.93	1.24 ± 1.07	0.44^a^
OSDI score	5.13 ± 5.37	6.34 ± 7.35	0.50^a^	5.25 ± 5.40	6.33 ± 7.48	0.66^a^
Adherence rate (%)	92.81 ± 7.49	91.38 ± 14.40	0.50	92.36 ± 7.70	93.92 ± 9.78	0.14
Drug tolerance score						
Stinging/burning	0.19 ± 0.48	0.28 ± 0.53	0.46	0.21 ± 0.50	0.29 ± 0.55	0.55
Sticky eye sensation	0.23 ± 0.50	0.21 ± 0.56	0.64	0.21 ± 0.50	0.25 ± 0.61	0.99
Itching	0.23 ± 0.56	0.48 ± 0.69	0.072	0.18 ± 0.48	0.42 ± 0.65	0.11
Blurred vision	0.32 ± 0.54	0.10 ± 0.41	**0.036**	0.32 ± 0.55	0.13 ± 0.45	0.084
Sandiness/grittiness	0.26 ± 0.51	0.34 ± 0.48	0.36	0.18 ± 0.39	0.33 ± 0.48	0.21
Dryness	0.26 ± 0.58	0.07 ± 0.26	0.15	0.14 ± 0.36	0.04 ± 0.20	0.23
Light sensitivity	0.10 ± 0.30	0.03 ± 0.19	0.35	0.07 ± 0.26	0.04 ± 0.20	0.67
Pain or soreness	0.10 ± 0.30	0.14 ± 0.44	0.91	0.11 ± 0.31	0.04 ± 0.20	0.39
Patient satisfaction score						
Easier to open	1.90 ± 0.91	1.21 ± 0.49	**< 0.001**	1.96 ± 0.92	1.25 ± 0.53	**0.002**
Easier for installation	2.26 ± 0.82	1.93 ± 1.03	0.11	2.29 ± 0.81	1.96 ± 1.00	0.15
Easier for storage	1.48 ± 0.57	1.28 ± 0.45	0.15	1.50 ± 0.58	1.25 ± 0.44	0.10
Convenient for drug management	1.55 ± 0.77	1.07 ± 0.26	**0.002**	1.57 ± 0.79	1.08 ± 0.28	**0.005**
Willingness for sustainable use of medication						
Yes, n (%)	26 (83.9)	21 (72.4)	0.28	25 (89.3)	20 (83.3)	0.69
No, n (%)	5 (16.1)	8 (27.6)		3 (10.7)	4 (16.7)	

*OSDI* ocular surface disease index.

^a^Statistical analyses are conducted using ANCOVA after adjusting baseline data.

Significant values with *P* < 0.05 are indicated in bold.

### Secondary outcomes

The preservative-free group showed a significantly lower corneal staining score compared to that in the preserved group at 4 weeks (Table 3). Additionally, the preservative-free group showed a better corneal staining score compared to that at baseline. In contrast, the conjunctival staining scores did not differ significantly between the preservative-free and preserved groups at 4 weeks, and neither group showed significant changes compared to the baseline values. At 4 weeks, both groups showed similar OSDI scores, which were significantly better than those at baseline. The TBUT did not differ significantly between the groups at 4 weeks; however, the preservative-free group showed a significantly longer TBUT than that in the preserved group at 12 weeks. Additionally, while the preserved group showed a significantly shorter TBUT at 12 weeks, no change was observed in the preservative-free group. For the PP set, the preservative-free group showed less bulbar hyperemia compared to the preserved group at 4 weeks but the degrees of hyperemic change were similar at 12 weeks. The preservative-free group showed less bulbar hyperemia at 12 weeks compared to baseline. However, no inter- and intragroup differences in limbal hyperemic change were observed at 4 and 12 weeks. Both groups showed significantly lower IOP at both 4 and 12 weeks compared to those at baseline, with no significant differences between the groups throughout the study period.

**Table 3:** Secondary outcome measurement at 4-week visit (V3) and 12-week visit (V4).

Variables	Intention-to-treat set			Per-protocol set		
	Preserved (n = 31)	Preservative-free (n = 29)	*P*	Preserved (n = 28)	Preservative-free (n = 24)	*P*
Corneal staining score (V3)	0.68 ± 0.75	0.21 ± 0.49	**0.003**	0.61 ± 0.74	0.21 ± 0.51	**0.011**
Conjunctival staining score (V3)	1.42 ± 0.87	1.36 ± 0.93	0.42	1.35 ± 0.85	1.31 ± 0.86	0.67
OSDI score (V3)	4.29 ± 6.91	5.55 ± 6.16	0.29	4.57 ± 7.20	5.71 ± 6.54	0.33
TBUT (V3), sec	6.40 ± 2.04	5.87 ± 1.71	0.19	6.30 ± 2.12	6.06 ± 1.76	0.53
TBUT (V4), sec	5.55 ± 1.74	6.86 ± 2.91	**0.035**	5.61 ± 1.72	7.27 ± 2.96	**0.017**
Hyperemic score						
Bulbar (V3)	1.03 ± 0.91	0.79 ± 0.68	0.11	0.96 ± 0.92	0.67 ± 0.56	**0.047**
Bulbar (V4)	1.00 ± 0.97	0.86 ± 0.83	0.64	0.86 ± 0.80	0.67 ± 0.64	0.37
Limbal (V3)	0.71 ± 0.78	0.66 ± 0.67	0.53	0.68 ± 0.77	0.58 ± 0.65	0.40
Limbal (V4)	0.77 ± 0.92	0.66 ± 0.72	0.69	0.61 ± 0.69	0.50 ± 0.66	0.56
IOP (V3), mmHg	13.42 ± 3.91	13.38 ± 2.80	0.45	13.54 ± 4.06	13.42 ± 2.98	0.25
IOP (V4), mmHg	12.87 ± 3.76	13.38 ± 2.70	0.077	12.89 ± 3.96	13.17 ± 2.81	0.14

*OSDI* ocular surface disease index, *TBUT* tear-film break up time, *IOP* intraocular pressure.

Comparative analyses were performed using ANCOVA after adjusting IOP, age, central corneal thickness, and baseline data.

Significant values with *P* < 0.05 are indicated in bold.

### Safety assessments

The BCVA did not differ between the groups at 4 and 12 weeks (Table 4). After 12 weeks, both groups showed similar levels of ocular symptoms, including stinging/burning, sticky eye sensation, itching, sandiness/grittiness, dryness, light sensitivity, and pain/soreness (Table 2). The preservative-free group showed less blurring of vision compared to the preserved group (*P* = 0.036, ITT set).

**Table 4:** Safety assessment at 4- and 12-week visits using per-protocol set.

Variables	4-week visit			12-week visit		
	Preserved (n = 31)	Preservative-free (n = 29)	*P*	Preserved (n = 31)	Preservative-free (n = 29)	*P*
BCVA	0.88 ± 0.20	0.96 ± 0.08	0.090	0.90 ± 0.19	0.94 ± 0.11	0.48
SBP, mmHg	132.65 ± 15.10	124.31 ± 14.41	0.058	131.00 ± 16.55	126.14 ± 16.43	0.37
SBP difference from baseline	− 4.93 ± 11.72	− 1.72 ± 11.02	0.37	− 6.03 ± 10.85	0.79 ± 12.81	**0.032**
DBP, mmHg	77.23 ± 8.92	73.38 ± 10.28	0.11	75.27 ± 9.73	76.04 ± 12.24	0.99
DBP difference from baseline	− 1.90 ± 9.95	− 0.52 ± 11.14	0.64	− 3.55 ± 7.85	2.68 ± 10.35	**0.021**
HR, bpm	73.10 ± 10.08	78.00 ± 11.25	0.26	75.73 ± 9.27	77.14 ± 12.08	0.65

*BCVA* best-corrected visual acuity, *SBP* systolic blood pressure, *DBP* diastolic blood pressure, *HR* heart rate.

Comparative analyses were performed using ANCOVA after adjusting age, gender, and baseline data, except for BCVA. For BCVA, Wilcoxon rank-sum test was used.

Significant values with *P* < 0.05 are indicated in bold.

The preservative-free group showed a significantly lower SBP at baseline compared to that in the preserved group; however, SBP, DBP, and HR did not differ significantly throughout the study period (Table 4). While the preserved group showed a significant reduction in SBP and DBP from baseline at 4 and 12 weeks, the preservative-free group showed stable SBP and DBP throughout all visits. The HR and physical examination results did not differ significantly within and between the groups. Two patients (6.5%) from the preserved group and four patients (13.8%) from the preservative-free group withdrew from the study due to adverse events (*P* = 0.42). The commonly reported adverse events associated with drug discontinuation were conjunctival hyperemia, allergic conjunctivitis, pruritis, and headache.

## Discussion

This prospective multicenter, investigator-masked study compared preservative-free and preserved brimonidine tartrate 0.15% in patients with open-angle glaucoma and ocular hypertension. During 12 weeks of follow-up, our results showed similar corneal and conjunctival staining and OSDI score for both types of drug. However, the preservative-free group showed better tear-film stability and less bulbar hyperemia at 12 weeks. The preservative-free group also showed stable hemodynamic values during the 12 weeks. Thus, preservative-free brimonidine tartrate showed comparable efficacy and safety, and higher patient satisfaction compared to preserved brimonidine tartrate.

Preservative-free formulations can improve the physiological state of the ocular surface and tear film by providing symptomatic relief and reducing preservative-induced corneal toxicity^7,8^. The present study showed similar corneal and conjunctival staining and OSDI score between the preservative-free and preserved groups after 12 weeks. Longer follow-up periods may be required for more accurate comparison; however, these findings imply the reduced toxicity of Purite^®^ compared to other traditional preservatives such as benzalkonium chloride, consistent with previous studies^11,17,18^. However, the corneal staining score was significantly lower in the preservative-free group than that in the preserved group at 4 weeks and was significantly better than the baseline score. Although such differences were not observed at 12 weeks, the preservative-free group still showed a longer TBUT compared to that in the preserved group. The difference in the other inactive ingredients between these formulations could have contributed to this difference. However, we speculate that our results may be more associated with the overall combination of the drug components. The active ingredient, brimonidine, can also be toxic to cells at clinically relevant concentrations, but the pharmacodynamics effect, toxicity, or the interaction with inactive ingredients can be determined within the overall drug formulation^19^. We believe that these factors were associated with the maintenance or improvement in the corneal tear film stability in the preservative-free group. Despite the lower toxicity of Purite, the preservative-free formulation may still have better effects on corneal tear films. Given these factors, the lower rate of blurred vision in the preservative-free group compared to that in the preserved group without any difference in burning sensation may also be related to the overall drug formulation, contrary to the findings of a previous study^20^.

Patient adherence is a major concern for glaucoma treatment as it significantly affects patient life-long medication use and overall disease progression. Although the overall adherence rates were similar between the groups, the preservative-free group showed higher patient satisfaction levels; these patients reported that the single-dose container was easy to open and convenient for drug management. Compared to the preserved group, approximately two-fold more patients in the preservative-free group reported that it was very easy to open the single-dose container. While the difficulty of opening rigid plastic containers of singe-dose units in elderly patients remains controversial^21,22^, this has not been considered a hurdle in this newly developed preservative-free formulation. Moreover, patients can easily count the number of used or unused doses with the single-dose container system or keep the drug in desired locations, leading to better drug accessibility, resulting in convenient drug management, another advantage of the preservative-free formulation in this study.

Previous studies reported generalized BP reduction after brimonidine use, with different results depending on the duration of use: a significant decrease in SBP and DBP was reported after short-term brimonidine use^23–25^, whereas no significant reduction was observed after its long-term use^26,27^. Brimonidine induced a significant decrease in SBP and DBP at all time points during a 24-h period^24^. Another study reported a significant decrease in SBP at 2 and 4 weeks but not at 8 and 12 weeks^27^. Our results showed lower SBP and DBP at 4 and 12 weeks compared to baseline measures when using brimonidine only in the preserved group. Even after comparing the amount of reduction by adjusting for age and sex, the results remained consistent. Although the preservative-free group showed decreased SBP and DBP at 4 weeks, these levels had recovered at 12 weeks. This may be attributable to differences in systemic absorption between the two drugs, which are affected by various factors, including inherent drug viscosity, individual nasolacrimal drainage rates, and individual drug consumption. The present study initially excluded patients with a prior history of lacrimal punctal occlusion procedures. While the status of the external punctal opening or the passage of the lacrimal drainage system may have differed between the groups, this information was lacking in our study population. Additionally, while the overall viscosities were similar between the drugs, subtle differences in the inactive ingredients may have decreased the systemic absorption of the preservative-free formulation, resulting in stable BP and HRs. The difference in systemic vascular properties of the patients in the two groups in response to the drug may have also contributed to the difference; however, the proportion of patients with systemic vascular disease or taking systemic medications did not differ significantly between the groups. Finally, another important variable may be the degree of individual drug consumption. Na et al. reported that drug over-consumption was significantly associated with bottle-type dispensers, while no association was found for unit-dose pipettes^28^. Furthermore, as the bottle of preserved brimonidine is not transparent, patients may have unintentionally instilled several drops at once. Further investigations using different study populations with more accurate data on such causative conditions may be required.

Regarding drug safety, both drugs showed similar rates of ocular adverse events leading to drug discontinuation. The well-known primary side effects associated with preserved brimonidine tartrate ophthalmic solutions are allergic conjunctivitis, blepharitis, and conjunctival hyperemia^1,29^. These are presumed to be more associated with the drug component itself than the preservative. Consistent with previous reports, approximately 10.0% of patients were withdrawn from the study due to the following mild adverse events: headache, allergic conjunctivitis, hyperemia, and pruritis in the preservative-free group and hyperemia and pruritis in the preserved group. However, no moderate to severe adverse events were observed in the present study.

Several limitations should be considered when interpreting our results. First, the follow-up period of 12 weeks may not be long enough for safety assessment, patient satisfaction, or adherence rates. Although our clinical trial has limitations in replicating the effect of years of topical eye drop use, this study has the longest follow-up period up reported to date. Second, this study did not include patients with severe corneal/conjunctival disease, who may have benefitted more from the preservative-free medication and showed different corneal surface assessment results. However, such a patient group might have required additional medication other than glaucoma drugs, which could mask the effect of our testing drugs. Further studies with patients having different corneal properties and longer follow-up may be required to investigate the long-term effect of preservative-free and preserved medications in diverse settings. Lastly, the present study required instillation three times per day. The results may have differed for twice-daily instillation of the drug, which is another prevalent trend of brimonidine use in South Korea. Nonetheless, our results showed better corneal tear film stability and patient satisfaction for preservative-free brimonidine tartrate, which may have more advantages in cases with higher numbers of instillations requiring better patient medication adherence.

In conclusion, the results of our multicenter, prospective, randomized clinical trial showed comparable corneal and conjunctival status and adherence rates for preservative-free brimonidine tartrate, with better corneal tear film stability after 12 weeks. Moreover, patient satisfaction level and stable hemodynamic parameters were better for preservative-free brimonidine tartrate compared to preserved brimonidine, indicating its potential advantages for patients requiring long-term use of glaucoma medication.

## Data Availability

The datasets used and/or analysed during the current study are available from the corresponding author on reasonable request.
